# A Comparison of Two Pain Scales in the Assessment of Dental Pain in East Delhi Children

**DOI:** 10.5402/2012/247351

**Published:** 2012-02-14

**Authors:** Amit Khatri, Namita Kalra

**Affiliations:** Department of Paedodontics and Preventive Dentistry, University College of Medical Sciences & GTB Hospital, University of Delhi, Delhi 110095, India

## Abstract

Pain is the most common symptom of oral diseases. Pain perception in children is highly variable and unreliable due to poor communication. Therefore we designed a study to compare pain measurement techniques, that is, visual analogue scale (VAS) and Wong-Baker faces pain rating scale (WBFPS) among Delhi children aged 3 to 14 years undergoing dental extraction. *Method*. A cross-sectional study was conducted on 180 patients aged 3 to 14 years who had undergone dental extraction. Children were assessed for their pain sensitivity using visual analogue scale (VAS) and Wong-Baker faces pain rating scale (WBFPS ). *Result and Conclusion*. Pain threshold tends to decline, and the self-management of pain becomes more effective with increasing age. Genderwise result shows that communication ability of boys and girls is similar in all age groups.

## 1. Introduction

Pain is referred to as the fifth vital sign and is an important reason for which patients seek health care [1].Scales to assess pain in children have been extensively studied [2]. But there are few pediatric studies to establish the validity of these tools in nonwestern cultures. Pain can be measured by self-report, biological markers, and behaviour because pain is subjective; self-report is the best if available [3]. Even though there are recommended guidelines for assessment of pain in children [4, 5], in India there is still limited data, on use of pain scale in children. It will be useful to know which pain assessment scale is more appropriate in Indian children. At the same time, there is need to evaluate how the health care professionals perceive the pain in children undergoing dental extraction. We have undertaken this study to compare the effectiveness of two pain scales in a dental setup both agewise as well as genderwise.

## 2. Aims and Objectives

The aim of this study is to assess pain in 3–14-year-old children in a dental setup and also to compare pain measurement techniques, that is, visual analogue scale (VAS) and Wong-Baker faces pain rating scale (WBFPS).

## 3. Materials and Method

### 3.1. Study Population

This was a cross-sectional study on 180 paediatric dental patients. The study was conducted in Department of Paedodontics and Preventive Dentistry, University College of Medical sciences & GTB Hospital (University of Delhi).

Inclusion Criteria: Children aged 3 to 14 years of east Delhi were included in the study for perception of pain after obtaining informed consent from parents.

Exclusion Criteria: Nonresidents of Delhi, physically disabled children, medically compromised children, and children who had no previous bad experience in dental clinics were excluded. Patients were divided into three groups on the basis of age:

Group I—3 to 6 years,
Group II—7 to 9 years,
Group III—10 to 14 years.

And each group is further divided on the basis of gender.

### 3.2. Data Collection

**Figure 1:**
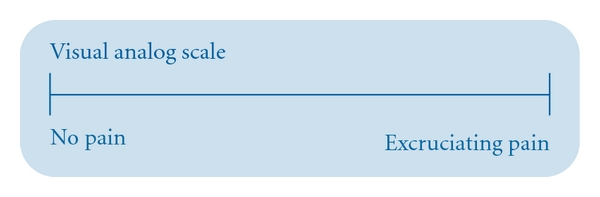
Visual analogue scale (VAS).

**Figure 2:**
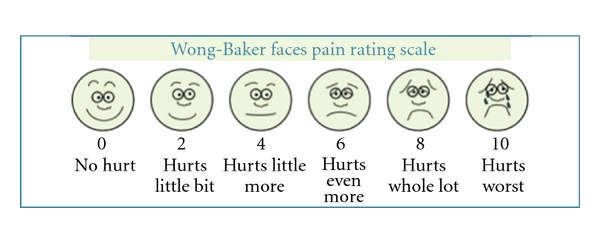
Wong-Baker faces pain rating scale (WBFPS).

 Data collection was one during a 7-month study while patients were sitting on dental chair after extraction. Each child was asked to grade present pain on visual analogue scale (VAS) [6] and Wong-Baker faces pain rating scale (WBFPS) [7] providing an evaluation of pain intensity at the moment of interview of patient. These scales were present sequentially. The visual analogue scale (VAS) is a line approximately 10 mm in length with each end anchored by extreme descriptive (e.g., no pain versus worst pain imaginable) (Figure 1). Patients were asked to make a mark on the line that represented their level of perceived pain intensity. Wong-Baker faces pain rating scale (WBFPS) presents 6 faces with increasing degree of pain from left to right. Each face was attributed scale from 0 to 10 indicated on scale. Children were asked to choose the face that best describe his or her own pain. Children were taught that each face is for a child who has no pain or some, or a lot. Face 0 does not hurt at all, Face 2 hurts just a little bit, Face 4 hurts a little more, Face 6 hurts even more, Face 8 hurts a whole lot, and Face 10 hurts as much as you can imagine (Figure 2).

## 4. Result

**Figure 3:**
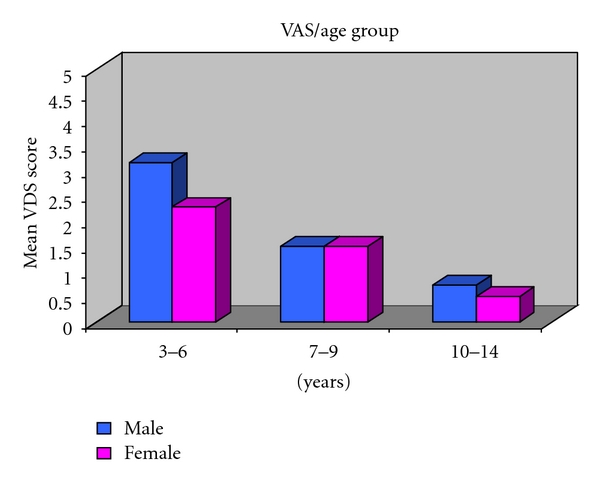
Mean VAS according to age group in both males and females.

The two pain scales were correlated with one another. Mean visual analogue scale (VAS) according to age in Group I in both males and females is 3.15 and 2.27, respectively. In Group II mean visual score is 1.5, respectively, for both males and females and for Group III mean visual score is 0.73 for males and 0.8 for females (Table 1). Analysis of subgroup showed that all scores were significantly correlated in both the sexes. On analysis by age, visual analogue scale (VAS) showed highly significant difference in score between Group I and Group III but not in Group I and Group II (Figure 3) ANOVA, *P* = 0.465 between the sex. Interaction between sex and agegroup is not significant (*P* = 0.751).

**Figure 4:**
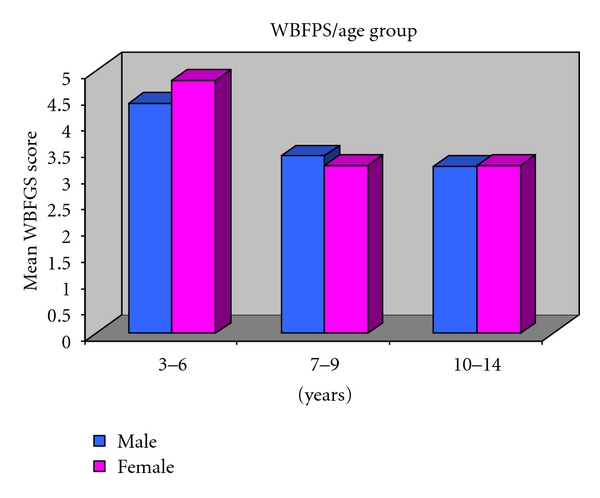
Mean WBFPS according to age group in both males and females.

Mean Wong-Baker faces pain rating scale (WBFPS) according to age in Group I in both males and females is 4.3 and 4.8, respectively. In Group II mean score is 3.3 and 3.2 for males and females, respectively, and for Group III mean score is 3.1 for males and 3.2 for females (Table 2).  Figure 4 depicts that Wong-Baker faces pain rating scale (WBFPS) showed highly significant difference in Group I and Group II and Group I and Group III, ANOVA, *P* = 0.823 between the sex. Interaction between sex and agegroup is not significant (*P* = 0.751).

**Figure 5:**
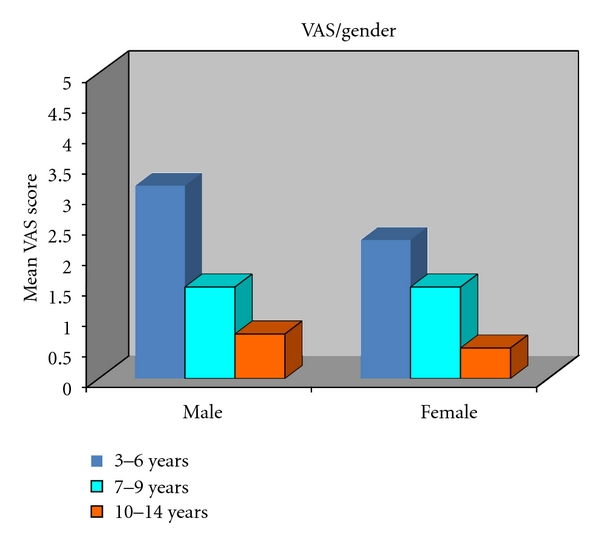
VAS according to gender in all groups.

**Figure 6:**
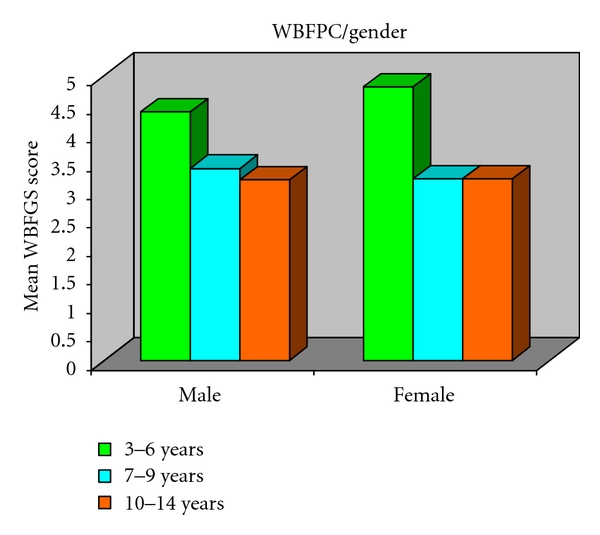
WBFPS according to gender in all groups.

Genderwise no statistical significant difference was evidenced in any age group in all the age groups both in case of visual analogue scale (VAS) (Figure 5) and Wong- Baker faces pain rating scale (WBFPS) (Figure 6).

For visual analogue scale (VAS) analysis of variance indicates *P* = 0.005 among the age groups; Posthoc Tukey's test depicts Group I is significantly different from Group III (*P* = 0.003). Interaction between sex and agegroup is not significant (*P* = 0.751). For Wong-Baker faces pain rating scale (WBFPS) analysis of variance indicates *P* = 0.005 among the age groups; Posthoc Tukey's test shows Group I is significantly different from Group II (*P* = 0.028) and Group III (*P* = 0.022). Interaction between sex and agegroup is not significant (*P* = 0.820).

## 5. Discussion

Most dentists are expert at handling children, but the recording of anxiety, fear, and pain experienced by the child will be an excellent communication tool. The present study finding supports the utility of obtaining child self-report of pain and shows that both visual analogue scale (VAS) and Wong-Baker faces pain rating scale (WBFPS) scale were appropriate tools used for assesment of pain among children aged 3 to 14 year who undergo selected procedure among Indian population. Developmental changes in response to painful stimuli occur early in infancy. In fact anticipatory fears of sharp object can be seen in children around 1 year of age [8]. As a child matures, develops a broader vocabulary, and witnesses a variety of environments, his or her ability to communicate feeling becomes increasingly sophisticated.

The pain threshold tends to decline, and the self-management of pain becomes more effective with increasing age [9]. In our study there is definite difference in severity of pain and discomfort between 3-to-6 yr-old patients compared to 7-to-9-year-old and also 10-to-14-year-old patients.

It may appear difficult to measure the degree of pain or discomfort in a young child, especially preschool children because of there level of cognitive and language development. The scales were expected to show some degree of correlation since the faces scale can be considered as visual analogue scale (VAS), and the fact the faces scale are closely related to one another. 

In our experience, children had more difficulty understanding the use of visual analogue scale (VAS) than that of Wong-Baker faces pain rating scale (WBFPS). Pain routinely is measured by visual analogue scale (VAS). Although these provide a useful method of describing pain experience, they do not assess the multidimensional nature of pain. More sophisticated measures include analysis of the sensory, affective, and cognitive components of pain.

 In our study genderwise result shows that communication ability of boys and girls is similar in all age groups. Interaction between sex and agegroup is not significant (*P* = 0.751) both for visual analogue scale (VAS) and for Wong-Baker faces pain rating scale (WBFPS) (*P* = 0.820).

The behaviour of a child worsens with increase in intensity of pain because children may not have a fully developed ability to recognize and interpret the physiological and cognitive manifestations of anxiety; measures of dental pain in children have tended to concentrate on the behavioral component of fear or have used nonverbal tools such as pictures.

 An essential and major part of handling and treating pediatric dental patients is centered around managing their fear, anxiety, and pain; hence recording of the same creates an important document. Pain reporting shoud become a part of daily history taking before extractions in children [10].

## 6. Conclusion

Wong-Baker faces pain rating scale (WBFPS) was found to be more sensitive as compared to visual analogue scale (VAS). Communication ability of boys and girls is similar in all age groups. Pain threshold tends to decline, and pain management becomes more effective with increasing age.

## Figures and Tables

**Table 1:** Mean visual analogue score according to age.

3–6 yrs		7–9 yrs		10–14 yrs	
Male	Female	Male	Female	Male	Female
3.15	2.27	1.5	1.5	0.73	0.8

**Table 2:** Mean WBFPS according to age group.

3–6 yrs		7–9 yrs		10–14 yrs	
Male	Female	Male	Female	Male	Female
4.38	4.82	3.38	3.2	3.18	3.2
